# Supplementary material for: Synergistic Inhibition of Methicillin-Resistant Staphylococcus aureus (MRSA) by Melaleuca alternifolia Chell (Tea Tree) and Eucalyptus globulus Labill. Essential Oils in Association with Oxacillin
Source: Antibiotics (Basel). 2023 May 3;12(5):846. doi: 10.3390/antibiotics12050846 (PMC10215920; doi:10.3390/antibiotics12050846)
Supplement: Supplementary file 1 [file antibiotics-12-00846-s001.zip › antibiotics-2346406-supplementary.pdf]

Article

Synergistic inhibition of methicillin-resistant *Staphylococcus aureus* (MRSA) by *Melaleuca alternifolia* Chell (Tea Tree) and *Eucalyptus globulus* Labill. Essential Oils in association with Oxacillin

Ramona Iseppi *, Carla Condò and Patrizia Messi

Department of Life Sciences, University of Modena and Reggio Emilia, Via Giuseppe Campi 287, 41125 Modena, Italy
* Correspondence: ramona.iseppi@unimore.it

Table S1. Qualitative and semi-quantitative analysis of *Malaleuca alternifolia* Chell and *Eucalyptus globulus* Labill. essential oils. Data are expressed as % relative peak area values ± standard deviation (SD).

	[a]Lit. RI	[b]Exp. RI	*Melaleuca alternifolia*	*Eucalyptus globulus*
α–thujene	928	926	0.88[c]	-
α–pinene	936	932	2.56±0.1	2.33±0.1
camphene	950	947	-	0.51[c]
sabinene	973	972	0.07[c]	-
β–pinene	978	975	0.73[c]	0.99[c]
β–myrcene	989	991	0.80[c]	0.83[c]
α–phellandrene	1004	1004	0.45[c]	0.23[c]
α–terpinene	1017	1017	8.89±0.2	0.09[c]
p-cymene	1024	1024	2.84±0.1	-
limonene	1029	1028	1.88±0.1	0.93[c]
1,8-cineole	1032	1030	2.35±0.1	58.07±0.2
cis-β-ocimene	1038	1039	-	0.21[c]
γ–terpinene	1060	1059	20.16±0.2	1.11[c]
linalool	1099	1100	0.49[c]	12.05±0.2
camphor	1143	1146	-	4.39±0.1
borneol	1166	1167	-	1.00[c]
terpinen-4-ol	1177	1179	43.29±0.2	0.66[c]
α–terpineol	1190	1192	2.99±0.1	0.71[c]
linalyl acetate	1263	1264	-	10.95±0.2
α–cubebene	1351	1353	0.06[c]	-
α–copaene	1376	1379	0.10[c]	-
α–gurjunene	1409	1414	0.32[c]	-
β–caryophyllene	1420	1426	0.32[c]	0.98[c]
aromadendrene	1440	1445	1.07[c]	-
α–humulene	1453	1460	0.09[c]	0.22[c]
allo-	1460	1467	0.47[c]	-

aromadendrene				
germacrene D	1481	1488	-	0.11[c]
α–selinene	1493	1496	0.14[c]	-
ledene	1495	1501	1.31[c]	-
δ-cadinene	1523	1530	1.03[c]	-
globulol	1582	1586	0.06[c]	-
caryophyllene oxide	1589	1593	0.19[c]	0.06[c]
viridiflorol	1591	1601	0.17[c]	-
**Total identified**			97.07	96.55

[a] Literature retention indices (HP-5 MS column); [b] Experimental retention indices (HP-5 ms column); [c] SD < 0.05.

1 2 3 4 5

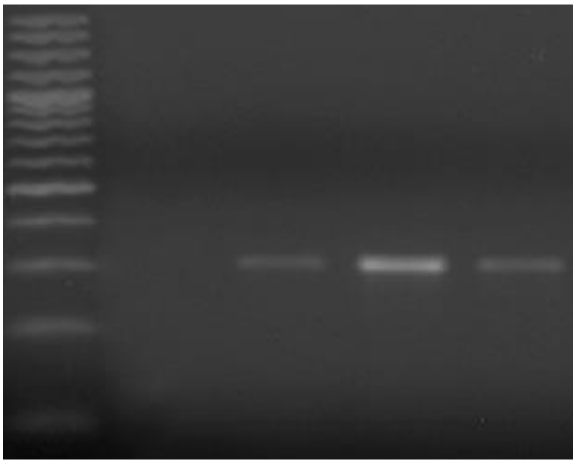

314 bp

Figure S1: Polymerase chain reaction (PCR) amplification of *mecA* gene. Lane 1: 100 bp DNA ladder, lane 2: *Staphylococcus aureus* ATCC 6538 (negative control), lane 3: *Staphylococcus aureus* 13, lane 4: *Staphylococcus aureus* 20, lane 5: *Staphylococcus aureus* 32.
